# Association of Bone Metastasis With Early-Stage Breast Cancer in Women With and Without Precancer Osteoporosis According to Osteoporosis Therapy Status

**DOI:** 10.1001/jamanetworkopen.2019.0429

**Published:** 2019-03-08

**Authors:** Hsiu-Man Chen, Fang-Ping Chen, Kang-Chung Yang, Shin-Sheng Yuan

**Affiliations:** 1 Institute of Biomedical Informatics, National Yang-Ming University, Taipei, Taiwan; 2 Bioinformatics Program, Taiwan International Graduate Program, Institute of Information Science, Academia Sinica, Taipei, Taiwan; 3 Institute of Statistical Science, Academia Sinica, Taipei, Taiwan; 4 Osteoporosis Prevention and Treatment Center, Department of Obstetrics and Gynecology, Keelung Chang Gung Memorial Hospital, Keelung, Taiwan; 5 College of Medicine, Chang Gung University, Taoyuan City, Taiwan; 6 Community Medicine Research Center, Keelung Chang Gung Memorial Hospital, Keelung, Taiwan

## Abstract

**Importance:**

Deaths from cancer are attributed more to secondary than primary tumors, but the pathogenesis of organ-specific cancer metastasis has not been determined.

**Objective:**

To investigate whether precancer osteoporosis and osteoporosis therapy are associated with alteration of bone metastasis patterns.

**Design, Setting, and Participants:**

This nationwide retrospective cohort study was performed from January 1, 2002, to December 31, 2013, using 2 cohorts from the Taiwan National Health Insurance Research Database: a random sample of 1 million beneficiaries from the Longitudinal Health Insurance Database who were enrolled in 2005 (LHID2005) and a specific data set of all the patients with osteoporosis. Patients diagnosed with breast cancer and precancer osteoporosis from January 1, 2002, to December 31, 2011, were included in the study, and their records were examined through December 31, 2013. From LHID2005, we selected 9104 women with early-stage breast cancer, of whom 705 had precancer osteoporosis. We identified 29 183 patients from the cohort of patients with breast cancer and osteoporosis, 14 020 of whom had precancer osteoporosis. Data analysis was performed from December 31, 2016, to August 31, 2018.

**Exposures:**

Precancer osteoporosis and osteoporosis therapy.

**Main Outcomes and Measures:**

The risk of bone metastasis in patients with and without precancer osteoporosis and patients receiving and not receiving osteoporosis therapy as well as time to bone metastasis development.

**Results:**

Among 9104 patients with breast cancer from the Longitudinal Health Insurance Database (mean [SD] age, 46.7 [14.0] years), precancer osteoporosis was not associated with a difference in risk of bone metastasis (adjusted hazard ratio [aHR], 0.87; 95% CI, 0.58-1.30; *P* = .49). Among 14 020 patients with precancer osteoporosis from the other cohort (mean [SD] age, 58.9 [11.6] years), osteoporosis therapy had no association with the risk of bone metastasis (bisphosphonates: aHR, 1.47; 95% CI, 1.00-2.17; *P* = .05; nonbisphosphonate drugs: aHR, 1.00; 95% CI, 0.72-1.39; *P* > .99). Compared with those without precancer osteoporosis (median time to bone metastasis development, 2.87 years; interquartile range [IQR], 1.34-4.86 years), among those with precancer osteoporosis, the median time to develop bone metastasis was shorter in those who did not receive treatment (1.74 years; IQR, 0.58-3.60 years; *P* < .001), whereas this time was the same for those who received treatment (bisphosphonates: 2.34 years; IQR, 1.23-3.13 years; nonbisphosphonate drugs: 2.08 years; IQR, 0.92-4.95 years).

**Conclusions and Relevance:**

Precancer osteoporosis was not associated with risk of bone metastasis, but untreated osteoporosis was associated with accelerated progression of bone metastasis when it occurred. Organ microenvironments interact with disseminated cancer mostly after the specific organ has been predetermined to be the designated location. Because recurrences and metastases are major obstacles to cancer treatments, determining which organs may be at risk for metastases may be crucial to treating the disease.

## Key Points

**Question:**

Is osteoporosis associated with the risk of bone metastasis in patients with breast cancer?

**Findings:**

In this cohort study using electronic medical records of 9104 patients with breast cancer and 14 020 patients with precancer osteoporosis, precancer osteoporosis was not associated with a difference in the incidence of bone metastasis and osteoporosis therapy was not associated with risk of bone metastasis. Untreated precancer osteoporosis was associated with accelerated secondary bone lesions.

**Meaning:**

The findings suggest that precancer osteoporosis is not associated with the risk of bone metastasis but untreated osteoporosis is associated with accelerated progression of bone metastasis when it occurs.

## Teaser

This cohort study investigates whether precancer osteoporosis and osteoporosis therapy are associated with bone metastasis patterns in patients with breast cancer.

## Introduction

Paget^1^ proposed the seed-and-soil hypothesis in 1889 through observing the distribution of disseminated cancer from 735 necropsies after breast cancer. Owing to research largely conducted in the 1970s and 1980s, we now know that disseminated tumor cells travel around the whole body but few can survive and form metastatic lesions. Therefore, to date, the best hypothesis to explain the phenomenon of organ-specific metastases is still the seed-and-soil theory.^2,3,4,5^

Although research that supports the seed-and-soil hypothesis emphasizes the interaction between tumor cells and the organ microenvironments, thus far little attention has been focused on whether different conditions of the host microenvironment would alter the patterns of secondary lesions (ie, the “soil”).^2,3,4,6,7^ In this study, we examined how different osseous microenvironments are associated with bone metastasis by looking at female patients with early-stage breast cancer with and without established osteoporosis.

Secondary malignant neoplasm of bone and bone marrow is a common complication of breast cancer and can occur in up to 80% of patients, and bone lesions in breast cancer are predominantly osteolytic.^8,9,10,11^ Osteolytic metastases are caused by osteoclast stimulation, which is mobilized by the increased levels of receptor activator of nuclear factor–κB ligand and inflammatory cytokines, such as interleukin 1 and tumor necrosis factor α.^6,8,9,11^

Such bone microenvironment mimics that of osteoporosis; thus, osteoporotic bones supposedly provide fertile premetastatic niches for disseminated cancer cells.^4,10,12^ As such, in this study, we used electronic medical records to examine whether precancer osteoporosis and osteoporosis therapy are associated with alteration of bone metastasis patterns.

## Methods

### Study Design and Cohorts

We conducted a nationwide retrospective cohort study using Taiwan’s National Health Insurance Research Database (NHIRD). The National Health Insurance (NHI) program was launched in 1995 as a compulsory program for all citizens in Taiwan and contains medical records of more than 99.9% of Taiwan’s 23 million population.^13,14,15^ Diagnosis and treatments of osteoporosis, breast cancer, and metastases are covered by the NHI. Cancer survivors undergo monitoring, including regular tumor marker tests and medical imaging; thus, the NHIRD contains the complete medical history of all the enrollees. This study followed the Strengthening the Reporting of Observational Studies in Epidemiology (STROBE) reporting guideline for cohort studies and has been approved by the institutional review boards of Academia Sinica and Chang Gung Medical Foundation. Informed consent was waived because only deidentified data were used.

The NHIRD service provided several types of data sets. In this study, we used 2 data sets. The first data set was a random sample of 1 million beneficiaries drawn from the Longitudinal Health Insurance Database (LHID) enrolled in 2005 (LHID2005). The second data set comprises the medical history of all patients with osteoporosis (BCOS) diagnosed between January 1, 1996, and December 31, 2011, in Taiwan. The sampling procedure of the database and the selection of the time frame are described in eMethods 1 in the Supplement.

### The LHID2005 Data Set

Figure, A shows the flow diagram of the LHID data set. After data cleaning, this data set contained 998 435 samples with electronic medical records for January 1, 1996, to December 31, 2013. From those 998 435 enrollees, we identified 9104 women who were diagnosed with early-stage breast cancer from January 1, 2002, to December 31, 2011, among whom 705 were diagnosed with osteoporosis before the diagnosis of breast cancer (exposed) and the other 8399 patients were not (unexposed). We retrieved the medical records of those 9104 patients from January 1, 2002, to December 31, 2013.

**Figure.**
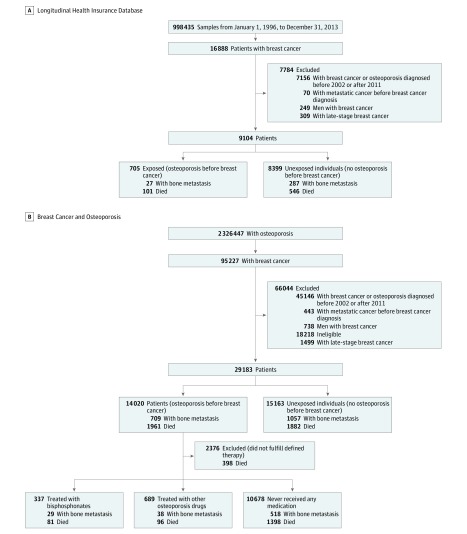
Flow Diagrams of the Study Cohorts Ineligible cases were those whose fracture events were caused by unintentional injuries. The definition of osteoporosis therapy is described in eMethods 3 in the Supplement. Sixty patients were treated with both types of therapy.

### The BCOS Data Set

The flow diagram of the BCOS data set is shown in Figure, B. This data set comprised 2 326 447 patients. We selected 29 183 women diagnosed with early-stage breast cancer from January 1, 2002, to December 31, 2011, of whom 14 020 had been diagnosed with osteoporosis before the diagnosis of breast cancer. We further identified which of these patients received osteoporosis therapy: 337 were treated with bisphosphonates, 689 were treated with other osteoporosis drugs, and 10 678 never received any medication before breast cancer diagnosis. We distinguished bisphosphonates from other osteoporosis drugs because bisphosphonates are widely used as the first-line therapy for osteoporosis.^16,17,18^ We also retrieved the records of these patients through December 31, 2013.

Basic characteristics of the 2 cohorts are given in Table 1, and definitions of breast cancer, osteoporosis, cancer staging, osteoporosis medication, and mortality are listed in eMethods 2 in the Supplement. The definition of osteoporosis therapy is described in eMethods 3 in the Supplement.

**Table 1.** Characteristics of the LHID2005 and BCOS Cohorts

Variable, Age Group	Osteoporosis Before Breast Cancer		*P* Value^a^
	Yes	No	
**Women With Early-Stage Breast Cancer in the LHID2005 Cohort**			
Age at breast cancer diagnosis, mean (SD), y			
Overall	58.9 (13.1)	45.7 (13.6)	<.001
>55 y	66.8 (9.2)	64.0 (7.6)	<.001
≤55 y	46.6 (7.8)	40.4 (9.8)	<.001
Radiation therapy, No./total No. (%)			
Overall	147/705 (20.9)	1394/8399 (16.6)	.005
>55 y	97/429 (22.6)	381/1890 (20.2)	.29
≤55 y	50/276 (18.1)	1013/6509 (15.6)	.29
Chemotherapy, No./total No. (%)			
Overall	182/705 (25.8)	1666/8399 (19.8)	<.001
>55 y	119/429 (27.7)	491/1890 (26.0)	.49
≤55 y	63/276 (22.8)	1175/6509 (18.1)	.05
Surgery, No./total No. (%)			
Overall	338/705 (47.9)	2844/8399 (33.9)	<.001
>55 y	241/429 (56.2)	944/1890 (49.9)	.02
≤55 y	97/276 (35.1)	1900/6509 (29.2)	.04
Metastasis, No./total No. (%)			
Bone	27/705 (3.8)	287/8399 (3.4)	.64
Lung	18/429 (4.2)	230/1890 (12.2)	.07
Liver	14/276 (5.1)	193/6509 (3.0)	.69
**Women With Early-Stage Breast Cancer in the BCOS Study Cohort**^b^			
Age at breast cancer diagnosis, mean (SD), y			
Overall	60.9 (11.7)	57.0 (11.3)	<.001
>55 y	66.9 (9.1)	66.0 (7.9)	<.001
≤55 y	49.0 (5.3)	47.9 (5.2)	<.001
Radiation therapy, No./total No. (%)			
Overall	2886/14 020 (20.6)	2788/15 163 (18.4)	<.001
>55 y	1974/9317 (21.2)	1343/7645 (17.6)	<.001
≤55 y	912/4703 (19.4)	1445/7518 (19.2)	.83
Chemotherapy, No./total No. (%)			
Overall	3423/14 020 (24.4)	2986/15 163 (19.7)	<.001
>55 y	2363/9317 (25.4)	1450/7645 (19.0)	<.001
≤55 y	1060/4703 (22.5)	1536/7518 (20.4)	.006
Surgery, No./total No. (%)			
Overall	6648/14 020 (47.4)	6020/15 163 (39.7)	<.001
>55 y	4913/9317 (52.7)	3336/7645 (43.6)	<.001
≤55 y	1735/4703 (36.9)	2684/7518 (35.7)	.19
**Women in the BCOS Cohort Who Received Osteoporosis Therapy Before Breast Cancer Diagnosis**^c^			
Bisphosphonates			
Age at breast cancer diagnosis, mean (SD), y			
Overall	73.0 (9.8)	59.8 (11.6)	<.001
>55 y	74.1 (8.7)	66.4 (8.9)	<.001
≤55 y	51.3 (3.3)	48.6 (5.5)	.05
Radiation therapy, No./total No. (%)	64/337 (19.0)	2233/10 678 (20.9)	.43
Chemotherapy, No./total No. (%)	62/337 (18.4)	2700/10 678 (25.3)	.005
Surgery, No./total No. (%)	167/337 (49.6)	5047/10 678 (47.3)	.44
Other osteoporosis drugs			
Age at breast cancer diagnosis, mean (SD), y			
Overall	64.2 (10.6)	59.8 (11.6)	<.001
>55 y	67.6 (9.2)	66.4 (8.9)	<.001
≤55 y	51.5 (3.2)	48.6 (5.5)	<.001
Radiation therapy, No./total No. (%)	124/689 (18.0)	2233/10 678 (20.9)	.07
Chemotherapy, No./total No. (%)	136/689 (19.7)	2700/10 678 (25.3)	.001
Surgery, No./total No. (%)	301/689 (43.7)	5047/ 10 678 (47.3)	.07

Abbreviations: BCOS, breast cancer and osteoporosis; LHID2005, Longitudinal Health Insurance Database 2005.

^a^ *P* values were derived from unpaired, 2-tailed *t* tests (age) or χ^2^ tests.

^b^ The group with no precancer osteoporosis in this cohort only includes women whose osteoporosis was diagnosed after the diagnosis of breast cancer and does not include those who never had a diagnosis of osteoporosis during the study period.

^c^ The definition of osteoporosis therapy is described in eMethods 3 in the Supplement.

### Statistical Analysis

We assessed whether precancer osteoporosis was associated with an increased risk of developing bone metastasis. We chose precancer osteoporosis because cancer and cancer therapies are greatly responsible for bone loss,^16,19,20^ thus making postcancer osteoporosis unsuitable for our study purpose. For the LHID2005 cohort, we calculated hazard ratios (HRs) with 95% CIs for developing bone metastasis using Cox proportional hazards regression models adjusted for age at cancer diagnosis, which was treated as a continuous variable.

We then used the BCOS cohort to compare patients with breast cancer and precancer osteoporosis who received osteoporosis therapy with those did not receive osteoporosis therapy. We used age-adjusted Cox proportional hazards regression models to calculate HRs with 95% CIs for developing secondary bone lesions. At menopause, the uncoupling of bone resorption and formation increases the likelihood of developing osteoporosis,^12,21^ and our data sets showed that the number of patients with precancer osteoporosis surpassed that of those with postcancer osteoporosis at the approximate age of 55 years at breast cancer diagnosis (eFigure 1 in the Supplement); therefore, we further stratified the data according to this age threshold for all Cox proportional hazards regression models that we performed. We verified the proportional hazards regression assumption with the Schoenfeld residuals test and graphically examined the plots of Schoenfeld residuals against time.

We further examined the time it took for the patients to develop bone metastases by using the BCOS cohort. Because the distribution of time to bone lesions was not gaussian, we performed Wilcoxon rank sum tests for these nonparametric comparisons. We used MySQL, version 5.5 (Oracle Corporation) and Python, version 2.7 (Python Software Foundation) to extract data from the NHIRD and then used Matlab, version R2017a (MathWorks Inc) and R, version 3.4 (R Foundation for Statistical Computing) with packages survival and survminer for data analysis. We used Draw.io (JGraph Ltd) to draw flow diagrams. All tests were 2-sided, and *P* < .05 was considered to be statistically significant. Data analysis was performed from December 31, 2016, to August 31, 2018.

## Results

### Characteristics of Study Cohorts

Baseline characteristics of the 9104 patients from the LHID2005 cohort (mean [SD] age, 46.7 [14.0] years) and the 29 183 patients from the BCOS cohort (mean [SD] age, 58.8 [11.6] years) are given in Table 1. Exposed patients were significantly older at cancer diagnosis than unexposed patients in both the LHID2005 and BCOS cohorts (LHID2005: mean [SD] age, 58.9 [13.1] years in the exposed group and 45.7 [13.6] years in the unexposed group; *P* < .001; BCOS: mean [SD] age, 60.9 [11.7] years in the exposed group and 57.0 [11.3] years in the unexposed group; *P* < .001 by unpaired, 2-tailed *t* tests). Because age is a known factor for cancer and osteoporosis, we included this variable in statistical models.

Overall, bone was most likely to be the first distant recurrence site in the LHID2005 cohort (exposed, 27 of 705 patients [4.2%]; unexposed, 287 of 8399 patients [3.4%]) followed by lung (exposed, 18 of 429 patients [2.6%]; unexposed, 230 of 1890 patients [12.2%]) and liver (exposed, 14 of 276 patients [5.1%]; unexposed, 193 of 6509 [3.0%]). The mean (SD) follow-up time was 6.82 (3.04) years for the LHID2005 cohort and 6.95 (3.13) years for the BCOS cohort.

### Association of Precancer Osteoporosis and Osteoporosis Therapy With Bone Metastasis

In univariable and age-adjusted Cox proportional hazards regression models, there were no significant differences in the risk of relapse of bone cancer between exposed and unexposed patients with cancer (Table 2). Patients with osteoporosis before the diagnosis of breast cancer did not have a higher HR for bone metastasis (adjusted HR [aHR], 0.87; 95% CI, 0.58-1.30; *P* = .49). Age at diagnosis was a significant risk factor for developing secondary bone lesions (aHR, 1.04; 95% CI, 1.03-1.04; *P* < .001), especially for those who developed breast cancer before the age of 55 years (aHR, 1.06; 95% CI, 1.04-1.08; *P* < .001). In addition, when a patient developed secondary lesions, bone was most likely to be the first distant recurrence site for both exposed and unexposed individuals (exposed, 23 [37.7%]; unexposed, 220 [40.6%]) (eTable in the Supplement).

**Table 2.** Cox Proportional Hazards Regression Models for the Risk of Developing Bone Metastases

Variable	Univariable Analysis		Multivariable Analysis^a^	
	HR (95% CI)	*P* Value	Adjusted HR (95% CI)	*P* Value
All women with early-stage breast cancer^b^				
Osteoporosis status	1.33 (0.89-1.97)	.16	0.87 (0.58-1.30)	.49
Age at breast cancer diagnosis	NA	NA	1.04 (1.03-1.04)	<.001
Age >55 y^c^				
Osteoporosis status	0.93 (0.57-1.51)	.76	0.93 (0.57-1.51)	.76
Age at breast cancer diagnosis	NA	NA	1.00 (0.98-1.02)	>.99
Age ≤55 y^d^				
Osteoporosis status	1.14 (0.56-2.32)	.71	0.82 (0.40-1.68)	.59
Age at breast cancer diagnosis	NA	NA	1.06 (1.04-1.08)	<.001

Abbreviations: HR, hazard ratio; NA, not applicable.

^a^ Models adjusted for age.

^b^ The exposed group included 27 of 705 patients (3.8%) with bone metastasis; the unexposed included 287 of 8399 patients (3.4%) with bone metastasis.

^c^ The exposed group included 19 of 429 patients (4.4%) with bone metastasis; the unexposed included 108 of 1890 patients (5.7%) with bone metastasis.

^d^ The exposed group included 8 of 276 patients (2.9%) with bone metastasis; the unexposed included 179 of 6509 patients (2.8%) with bone metastasis.

Table 3 gives the results of the Cox proportional hazards regression models for the risk of relapse of bone marrow metastasis in patients with breast cancer and precancer osteoporosis who received and did not receive osteoporosis therapy. There was no significant difference in the risk of bone metastasis relapse between patients treated with nonbisphosphonate drugs and those who did not receive any treatment (aHR, 1.00; 95% CI, 0.72-1.39; *P* > .99) or those who were treated with bisphosphonates (aHR, 1.47; 95% CI, 1.00-2.17; *P* = .05). Model diagnostics based on Schoenfeld residuals showed that the Cox proportional hazards regression assumption was not violated in all the Cox proportional hazards regression models we performed (eFigure 2 in the Supplement).

**Table 3.** Stratified Univariable and Multivariable Analysis of Hazard Ratios for Developing Bone Metastasis

Variable	Univariable Analysis		Multivariable Analysis^a^	
	HR (95% CI)	*P* Value	Adjusted HR (95% CI)	*P* Value
Bisphosphonates^b^				
Osteoporosis treatment	2.01 (1.39-2.93)	<.001	1.47 (1.00-2.17)	.05
Age at breast cancer diagnosis	NA	NA	1.02 (1.02-1.03)	<.001
Age >55 y^c^				
Osteoporosis treatment	1.64 (1.11-2.43)	.01	1.46 (0.98-2.18)	.06
Age at breast cancer diagnosis	NA	NA	1.02 (1.00-1.03)	.008
Age ≤55 y^d^				
Osteoporosis treatment	0.84 (0.37-1.91)	.68	0.83 (0.36-1.89)	.65
Age at breast cancer diagnosis	NA	NA	1.00 (0.98-1.02)	.70
Other osteoporosis drugs^e^				
Osteoporosis treatment	1.10 (0.79-1.53)	.56	1.00 (0.72-1.39)	>.99
Age at breast cancer diagnosis	NA	NA	1.03 (1.02-1.03)	<.001
Age >55 y^f^				
Osteoporosis treatment	1.05 (0.73-1.51)	.80	1.03 (0.72-1.48)	.87
Age at breast cancer diagnosis	NA	NA	1.02 (1.01-1.03)	<.001
Age ≤55 y^g^				
Osteoporosis treatment	1.09 (0.53-2.22)	.81	1.09 (0.53-2.22)	.81
Age at breast cancer diagnosis	NA	NA	1.00 (0.98-1.02)	.74

Abbreviations: HR, hazard ratio; NA, not applicable.

^a^ Models adjusted for age.

^b^ The exposed group included 29 of 337 patients (8.6%) with bone metastasis; the unexposed group included 518 of 10 678 patients (4.8%) with bone metastasis.

^c^ The exposed group included 27 of 321 patients (8.4%) with bone metastasis; the unexposed group included 370 of 6711 patients (5.5%) with bone metastasis.

^d^ The exposed group included 2 of 16 patients (12.5%) with bone metastasis; the unexposed group included 148 of 3967 patients (3.7%) with bone metastasis.

^e^ The exposed group included 38 of 689 patients (5.5%) with bone metastasis; the unexposed group included 518 of 10 678 patients (4.8%) with bone metastasis.

^f^ The exposed group included 32 of 541 patients (5.9%) with bone metastasis; the unexposed group included 370 of 6711 patients (5.5%) with bone metastasis.

^g^ The exposed group included 6 of 148 patients (4.1%) with bone metastasis; the unexposed group included 148 of 3967 patients (3.7%) with bone metastasis.

### Association of Untreated Osteoporosis With Secondary Bone Lesions

Table 4 compares the time it took to develop secondary bone lesions between patients with vs without osteoporosis before breast cancer. Patients with precancer osteoporosis developed bone cancer relapse approximately 1 year quicker than those without precancer osteoporosis (median time, 1.78 [interquartile range (IQR), 0.60-3.48] years vs 2.87 [IQR, 1.34-4.86] years; *P* < .001). Detailed comparisons of time to bone metastases for patients with breast cancer with different osteoporosis statuses are shown in eFigure 3 in the Supplement.

**Table 4.** Comparisons of Time to Bone Metastasis

Characteristic	Median Time (IQR), y		Wilcoxon Rank Sum Test *P* Value
	Osteoporosis Before Breast Cancer	No Osteoporosis Before Breast Cancer	
Age at breast cancer diagnosis			
Overall, y	1.78 (0.60-3.48)	2.87 (1.34-4.86)	<.001
>55 y	1.74 (0.56-3.14)	2.81 (1.16-4.60)	<.001
≤55 y	1.97 (0.66-4.28)	3.05 (1.48-5.13)	<.001
Treatment			
Bisphosphonates	2.34 (1.23-3.13)	2.87 (1.34-4.86)	.08
Other osteoporosis drugs	2.08 (0.92-4.95)	2.87 (1.34-4.86)	.18
No therapy	1.74 (0.58-3.60)	2.87 (1.34-4.86)	<.001

Abbreviation: IQR, interquartile range.

When bisphosphonates were distinguished from other osteoporosis drugs, there were no differences in median time to bone metastasis from those without precancer osteoporosis (bisphosphonates: 2.34 [IQR, 1.23-3.13] years vs 2.87 [IQR, 1.34-4.86] years; *P* = .08; nonbisphosphonate drugs: 2.08 [IQR, 0.92-4.95] years vs 2.87 [IQR, 1.34-4.86] years; *P* = .18). However, among those who were never prescribed any medicine before cancer diagnosis, the time it took to develop bone metastasis was shorter (1.74 [IQR, 0.58-3.60] years vs 2.87 [IQR, 1.34-4.86] years; *P* < .001).

## Discussion

Breast cancer cells tend to form osteolytic metastases in bone,^8,9,10^ and the mechanism for forming osteolytic lesions has been well documented. Similar to osteoporosis, osteolytic metastases are caused by osteoclast stimulation and not by the direct effects of tumor invasion into bone.^8,9^ Osteoclast-activating factors, such as parathyroid hormone–related protein and interleukin 11, are released by tumor cells, which then promote the secretion of receptor activator of nuclear factor–κB ligand, which in turn induces the formation of osteoclasts and increases bone resorption.^6,8,9,10,12^

Such increased bone resorption in the osseous microenvironment has the same mechanism as osteoporosis, and studies^10,22,23^ have found that antiresorptive agents alleviated tumor burden in bone; in addition, bone resorption inhibitors, such as bisphosphonates, have been the standard of care for patients with bone lesions. As such, patients with precancer osteoporosis presumably provide more fertile premetastatic niches compared with those without precancer osteoporosis because of the number of osteoclast-mobilizing factors.

However, our study of a random sample of 9104 women with early-stage breast cancer found that those with precancer osteoporosis did not exhibit a greater risk of forming bone lesions. We then examined whether precancer osteoporosis therapy would make any difference to the results and found that it was also not associated with a difference; those treated with nonbisphosphonate drugs had near-identical risks to those without any treatment, and the association for bisphosphonates use was nonsignificant as well.

Age at cancer diagnosis was a significant risk factor for developing bone metastasis in our data, although other studies^24,25,26^ have reported it as protective against several types of distant recurrences for breast cancer. Although we treated age as a continuous variable, others^24,25,26^ divided it into several age groups and included some covariates, which might have contributed to such different results. In our data, the proportions of patients with bone lesions increased with age, as did the percentages of patients with distant metastases and those who died (eFigure 4 in the Supplement).

When we assessed the time it took for patients with breast cancer to develop bone metastasis among those in whom it developed, patients with precancer osteoporosis treated with either type of osteoporosis therapy did not have significant differences compared with those without precancer osteoporosis. However, those who never received osteoporosis therapy before the diagnosis of breast cancer developed bone lesions more quickly. In other words, untreated osteoporosis was associated with accelerated secondary bone lesions, and we suspect osteoporosis therapy only delayed tumor invasion into bone but did not prevent it.

In addition, our investigation did not support the “mechanical” hypothesis that proposed cancer cells spread through hematogenous or lymphogeneous metastatic patterns as a result of the anatomical structure.^2,4^ Fidler’s research using ^125^I-5-iodo-2′-deoxyuridine–labeled B16 melanoma cells has demonstrated that “when a plant goes to seed, its seeds are carried in all directions,”^1,5^ and we found that, in addition to bone and bone marrow, lung and liver were also often first sites of distant relapse (bone, 243 [40.3%]; lung, 158 [26.2%]; liver, 123 [20.4%]) (eTable in the Supplement). Because anatomical structures in humans are presumably similar, it is unlikely that tumor cells from the primary site would travel to different first destinations.

Our findings appear to contradict the seed-and-soil hypothesis of Paget,^1^ which has been the predominant explanation of nonrandom metastatic patterns, but that hypothesis had little definitive proof from clinical observation or empirical studies. Had the hypothesis been true, cancers that affect large numbers of postmenopausal patients would have relapsed in bone, but ovarian cancer cells seldom metastasize outside the peritoneal cavity and the most common distant recurrence site for endometrial carcinoma is the lung.^4,27,28^ In addition, in a mouse model that used the *W/W^v^* strain, a strain that has intrinsic defects in the bone marrow, to study how the change of the organ microenvironment would affect metastatic patterns, the incidence of *W/W^v^* in mice with bone lesions was not changed; only the mean number of colonized bones per mouse was different from their normal congenic controls.^2,7,29,30^

This latter example indicates that the defected bone marrow did not attract more metastasis, but the progression of metastatic tumor was different from that in healthy controls after the metastasis developed, which is comparable to our finding. These results suggest that organ microenvironments interact with disseminated cancer mostly after the specific organ has been predetermined to be the designated location. Although we are not able to determine the organ selection mechanism for metastasis, we suggest 2 conclusions from the gathered evidence. First, each cancer type has its preferred secondary lesion sites, but not every patient followed this preference. Second, the preferred metastatic site is not dictated by the microenvironment of the selected organ. We therefore conceived a hypothesis that metastatic sites had been predetermined by an unknown mechanism we called the *ecosystem of the body* before the seed landed on the soil, then the microenvironment of the soil supported the growth of the seeds after their landing.

### Limitations

This study has some limitations. First, the data sets lack some clinical variables, including cancer subtypes and tumor size, although research indicates that rates of bone lesions were similar among different subtypes of breast cancer, and the poor prognosis of triple-negative type was attributable to the excess risk of visceral metastases.^31^ Second, although the overall sample sizes were adequate, the small proportions of patients with bone metastases restricted our further investigation. Third, the osteoporosis cohort lacks patients who had never developed osteoporosis throughout the study period. Fourth, osteoporosis may be present for years before it is diagnosed; therefore, the associations might be underestimated.

## Conclusions

Our study found that osteoporosis that developed before breast cancer was not associated with the probability of forming secondary bone lesions, but untreated osteoporosis was associated with accelerated development of bone metastases should they occur; this finding suggests that organs of secondary lesions had been predetermined. Sites of distant metastases might be determined by the body rather than the organ microenvironments. Because recurrences and metastases are major obstacles to cancer treatments, determining the organ preferences for metastases may be crucial to treating the disease.

## Notes

Supplement. **eMethods 1.** Sampling Procedure of the National Health Insurance Research Database and Selection of a Diagnosis Window for the Data Sets **eMethods 2.** Definitions of Breast Cancer, Osteoporosis, Cancer Staging, Osteoporosis Medication, and Mortality **eMethods 3.** Definition of Osteoporosis Therapy **eFigure 1.** Age Distributions of the Studied Cohorts **eFigure 2.** Model Diagnostics for Cox Proportional Hazards Models Based on Schoenfeld Residuals **eFigure 3**. Plots for Comparisons of Time to Bone Metastases for Patients with Breast Cancer With Different Osteoporosis Statuses **eFigure 4.** Percentages of Patients With Metastases or who Died by Age **eTable.** Proportions of Patients Whose First Distant Metastatic Site was Bone, Lung, or Liver **eReferences**
