# Supplementary material for: Association of Bone Metastasis With Early-Stage Breast Cancer in Women With and Without Precancer Osteoporosis According to Osteoporosis Therapy Status
Source: JAMA Netw Open. 2019 Mar 8;2(3):e190429. doi: 10.1001/jamanetworkopen.2019.0429 (PMC6484629; doi:10.1001/jamanetworkopen.2019.0429)
Supplement: Supplement. — eMethods 1. Sampling Procedure of the National Health Insurance Research Database and Selection of a Diagnosis Window for the Data Sets eMethods 2. Definitions of Breast Cancer, Osteoporosis, Cancer Staging, Osteoporosis Medication, and Mortality eMethods 3. Definition of Osteoporosis Therapy eFigure 1. Age Distributions of the Studied Cohorts eFigure 2. Model Diagnostics for Cox Proportional Hazards Models Based on Schoenfeld Residuals eFigure 3. Plots for Comparisons of Time to Bone Metastases for Patients with Breast Cancer With Different Osteoporosis Statuses eFigure 4. Percentages of Patients With Metastases or who Died by Age eTable. Proportions of Patients Whose First Distant Metastatic Site was Bone, Lung, or Liver eReferences [file jamanetwopen-2-e190429-s001.pdf]

Supplementary Online Content

Chen H-M, Chen F-P, Yang K-C, Yuan S-S. Association of bone metastasis with early-stage breast cancer in women with and without precancer osteoporosis according to osteoporosis therapy status. *JAMA Netw Open.* 2019;2(3):e190429. doi: 10.1001/jamanetworkopen.2019.0429

eMethods 1. Sampling Procedure of the National Health Insurance Research Database and Selection of a Diagnosis Window for the Data Sets
eMethods 2. Definitions of Breast Cancer, Osteoporosis, Cancer Staging, Osteoporosis Medication, and Mortality
eMethods 3. Definition of Osteoporosis Therapy
eFigure 1. Age Distributions of the Studied Cohorts
eFigure 2. Model Diagnostics for Cox Proportional Hazards Models Based on Schoenfeld Residuals
eFigure 3. Plots for Comparisons of Time to Bone Metastases for Patients with Breast Cancer With Different Osteoporosis Statuses
eFigure 4. Percentages of Patients With Metastases or who Died by Age
eTable. Proportions of Patients Whose First Distant Metastatic Site was Bone, Lung, or Liver
eReferences

This supplementary material has been provided by the authors to give readers additional information about their work.

© 2019 Chen H-M et al. *JAMA Network Open.*

eMethods 1. Sampling Procedure of the National Health Insurance Research Database and Selection of a Diagnosis Window for the Data Sets

The National Health Insurance Research Database (NHIRD) service assigned each individual in the database a consecutive number, and then used Oracle's DBMS_RANDOM package (Oracle Corporation) to generate 1 million random numbers in order to draw 1 million random samples from the population. There were 22 717 053 beneficiaries in the database enrolled in the National Health Insurance (NHI) program in the year 2005. The 1 million samples drawn from this population are called the Longitudinal Health Insurance 2005 (LHID2005) cohort.

Although the International Classification of Diseases, Ninth Revision (ICD-9) system was used in large hospitals in Taiwan long before the year 1991 and was officially adopted by Taiwan's medical system in March 1991, the A-Code system was phased out in the year 2002, and some hospitals might still have been using the A-Code system before 2002. In the A-Code system, all metastases (ICD-9 codes: 196–198) were coded as A139; therefore, we selected the 2002-2011 diagnosis window to distinguish the locations of metastases and to have at least 2 years of follow-up time.

© 2019 Chen H-M et al. *JAMA Network Open.*

eMethods 2. Definitions of Breast Cancer, Osteoporosis, Cancer Staging, Osteoporosis Medication, and Mortality

Breast Cancer

ICD-9 codes: 174, 2330, 2383, and 2393.

Osteoporosis

ICD-9 codes: 7330, 805, 806, 808, 812, 813, 73312, 73313, and 73314; excluding fracture events caused by accidents: E81, E82, E83, E84, and E88.

Cancer Staging

The ICD-9 code 174 does not specify the staging of breast cancer; therefore, we included information of “secondary and unspecified malignant neoplasm” (ICD-9 codes: 196, 197, and 198) and selected those with no secondary and unspecified malignant neoplasm at cancer diagnosis as patients with early stage breast cancer.

Osteoporosis Medication

As for osteoporosis medicine, we included 5 bisphosphonates: alendronate, etidronate, ibandronic, pamidronate, and zoledronic acid; and 5 non-bisphosphonate drugs: calcitonin, denosumab, estrogen, raloxifene, and teriparatide. Risedronate and bazedoxifene were not prescribed in Taiwan from 2002 to 2013.

Mortality

As enrollment in the NHI program is compulsory for Taiwanese citizens, withdrawal from the program is often due to death of the enrollee.[1,2] However, we followed a more cautious procedure and included 5 additional criteria to define mortality. First, we included mortality information from the Catastrophic Illness Program, if the patient was enrolled in this program. Second, discharge reasons for hospitalized patients were death, suicide, or critical illness. Third, the patient was voluntarily discharged from the hospital against medical advice and then was removed from the NHI program in 28 days. Fourth, the patient was critically ill in the emergency department and received cardiopulmonary resuscitation, electrical defibrillation or cardioversion, endotracheal tube insertion, or epinephrine or norepinephrine injection and then was removed from the NHI program in 28 days. Fifth, we further required those patients who met the aforementioned criteria to have no visits and treatments in their medical records after NHI disenrollment.

© 2019 Chen H-M et al. *JAMA Network Open.*

eMethods 3. Definition of Osteoporosis Therapy

Osteoporosis therapy is defined as a treatment started before the diagnosis of breast cancer with at least 180 days' use of osteoporosis drugs in 1 year; the quantities of drugs were calculated according to the WHO's Defined Daily Dose system. We employed a dynamic programming algorithm to determine the medical adherence to osteoporosis therapy.

The "no therapy" group comprises those who had established osteoporosis before the diagnosis of breast cancer but were never prescribed any medicine before breast cancer diagnosis.

© 2019 Chen H-M et al. *JAMA Network Open.*

eFigure 1. Age Distributions of the Studied Cohorts

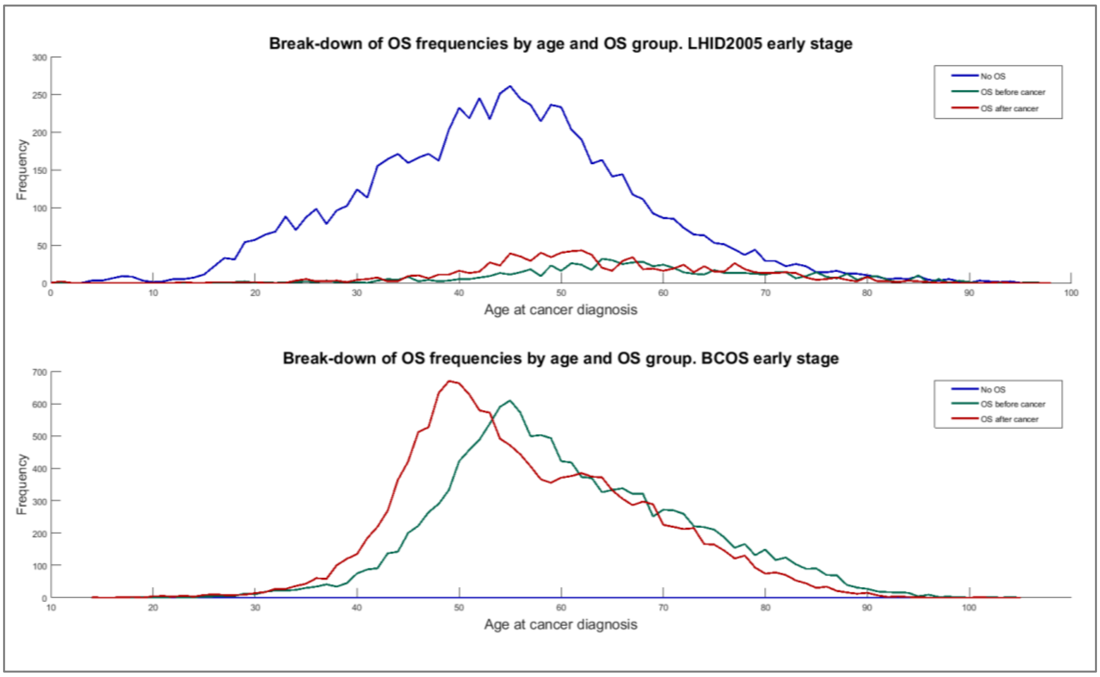

OS = osteoporosis.

© 2019 Chen H-M et al. *JAMA Network Open.*

eFigure 2. Model Diagnostics for Cox Proportional Hazards Models Based on Schoenfeld Residuals

(A) Age-adjusted Cox models for the risk of developing bone metastasis (LHID2005). (B) Age-adjusted Cox models, bisphosphonate users versus those never treated with any drug. (C) Age-adjusted Cox models, non-bisphosphonate users versus those never treated with any drug.

eFigure 2(A)

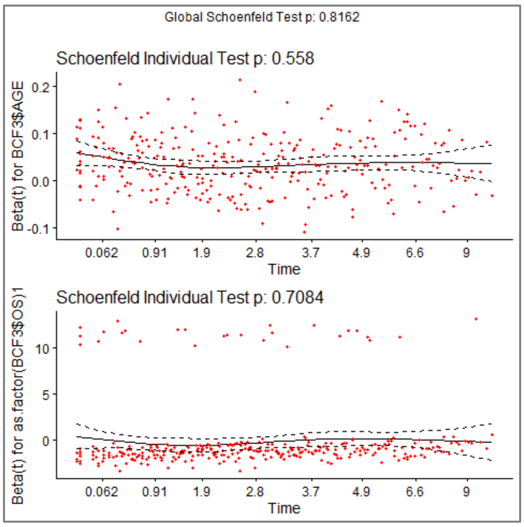

eFigure 2(B)

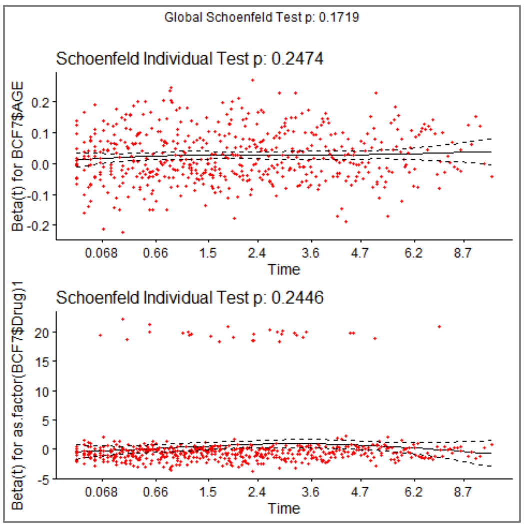

eFigure 2(C)

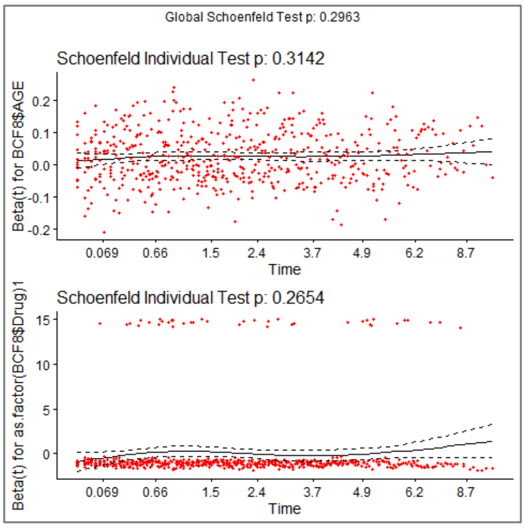

© 2019 Chen H-M et al. *JAMA Network Open.*

eFigure 3. Plots for Comparisons of Time to Bone Metastases for Patients With Breast Cancer With Different Osteoporosis Statuses

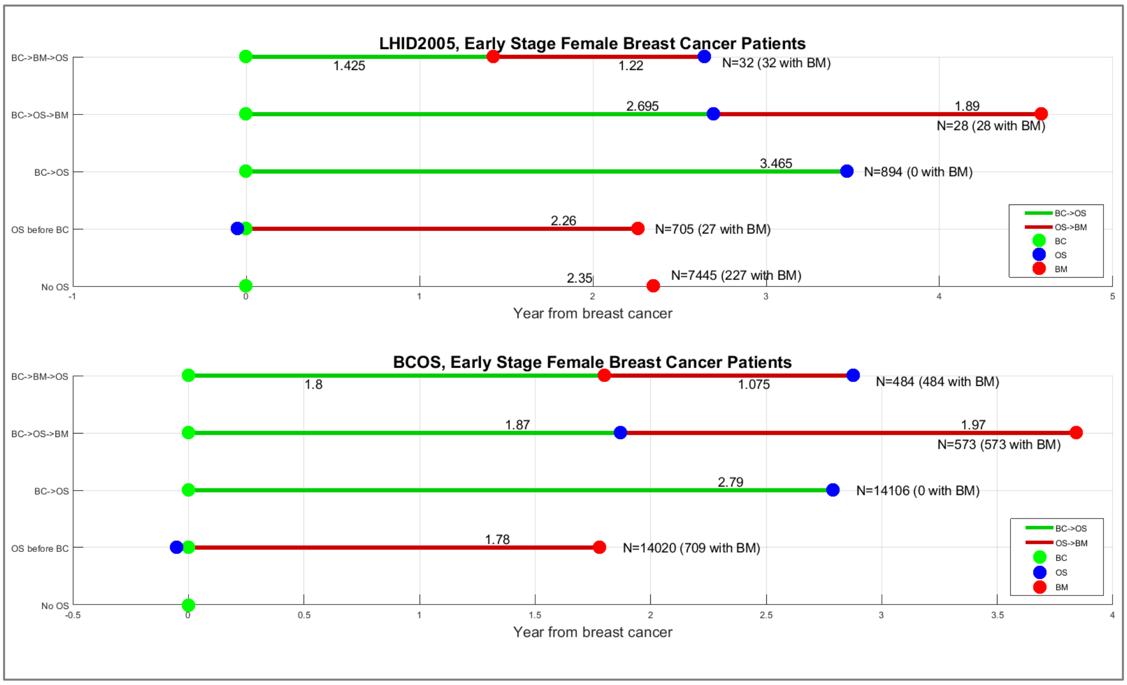

BC = breast cancer, OS = osteoporosis, BM = bone metastasis, N = sample size.

© 2019 Chen H-M et al. *JAMA Network Open.*

eFigure 4. Percentages of Patients With Metastases or who Died by Age

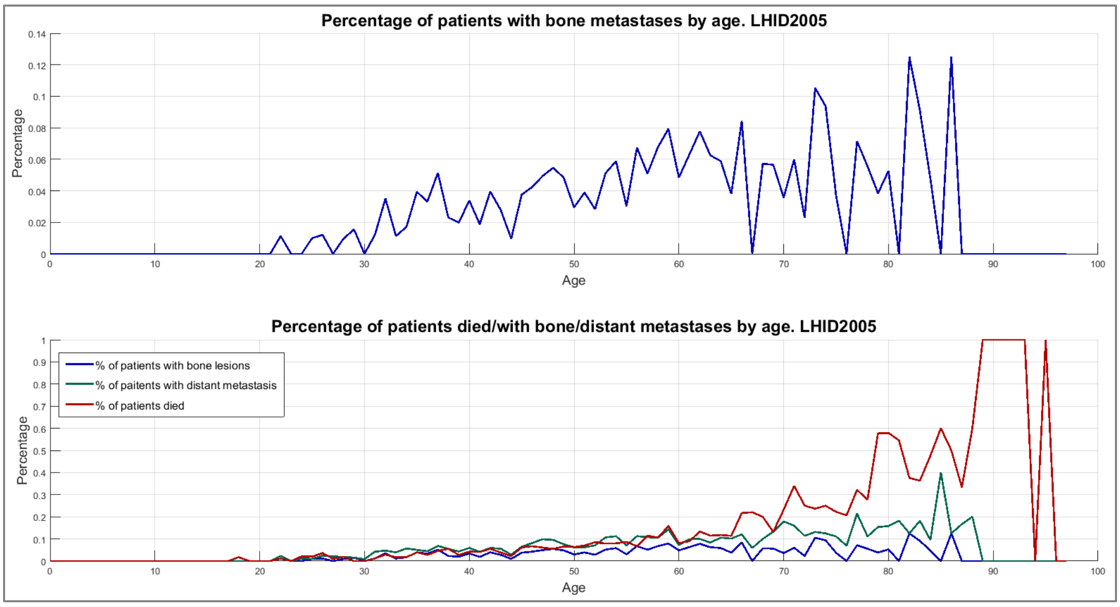

Age = age at breast cancer diagnosis.

© 2019 Chen H-M et al. *JAMA Network Open*.

eTable. Proportions of Patients Whose First Distant Metastatic Site was Bone, Lung, or Liver

Cohort	Stratum	Bone/Marrow	Lung	Liver	Total[a]
LHID2005	**Exposed**	23 (37.7%)	15 (24.6%)	8 (13.1%)	61
	**Unexposed**	220 (40.6%)	143 (26.4%)	115 (21.2%)	542
	**All**	243 (40.3%)	158 (26.2%)	123 (20.4%)	603

Results are expressed as N, or n. (%). Bone and bone marrow were most likely to be the first sites of distant metastasis for both exposed and unexposed groups.

[a] Total number of patients with any type of metastasis.

© 2019 Chen H-M et al. *JAMA Network Open.*

eReferences.

1. Wu C-Y, Chen Y-J, Ho H-J, Hsu Y-C, Kuo K-N, Wu M-S, Lin J-T. Association between nucleoside analogues and risk of hepatitis B virus-related hepatocellular carcinoma recurrence following liver resection. *JAMA*. 2012; 308(18): 1906-1913.

2. Cheng C-L, Chien H-C, Lee C-H, Lin W-J, Yang Y-H. Validity of in-hospital mortality data among patients with acute myocardial infarction or stroke in National Health Insurance Research Database in Taiwan. *International Journal of Cardiology*. 2015; 201: 96-101.

© 2019 Chen H-M et al. *JAMA Network Open.*
